# Social brains and divides: the interplay between social dominance orientation and the neural sensitivity to hierarchical ranks

**DOI:** 10.1038/srep45920

**Published:** 2017-04-05

**Authors:** Romain Ligneul, Romuald Girard, Jean-Claude Dreher

**Affiliations:** 1 Neuroeconomics, Reward and Decision-making Team, Institut des Sciences Cognitives Marc Jeannerod, Centre National de la Recherche Scientifique, 69675 Bron, France; 2 University Claude Bernard Lyon, Lyon 1, France

## Abstract

Ubiquitous in the animal kingdom, dominance hierarchies emerge through social competition and underlie the control of resources. Confronting the disruptive influence of socioeconomic inequalities, human populations tend to split into groups who legitimize existing dominance hierarchies and groups who condemn them. Here, we hypothesized that variations in the neural sensitivity to dominance ranks partly underpins this ideological split, as measured by the social dominance orientation scale (SDO). Following a competitive task used to induce dominance representations about three opponents (superior, equal and inferior), subjects were passively presented the faces of these opponents while undergoing fMRI. Analyses demonstrated that two key brain regions, the superior temporal sulcus (STS) and anterior dorsolateral prefrontal cortex (aDLPFC) were sensitive to social ranks. Confirming our hypothesis, the sensitivity of the right aDLPFC to social ranks correlated positively with the SDO scale, which is known to predict behaviors and political attitudes associated with the legitimization of dominance hierarchies. This study opens new perspectives for the neurosciences of political orientation and social dominance.

Social dominance is a natural and widespread phenomenon extending to most social species, including humans. However, unlike other animals, humans possess the unique ability to determine the social structures in which they live through the development of cultural, economic, linguistic or moral norms. Thus, human dominance hierarchies constitute a heavily debated construct in the political domain. On the one hand, the physiological and psychological stress endured by subordinate individuals^1^,^2^,^3^,^4^ leads an important part of the world population to wish the attenuation of dominance hierarchies. On the other hand, in many countries, democratic elections persistently demonstrate that their legitimization and the desire to reinforce them are also very prevalent. Several important demographic factors such as sex, ethnicity, income and education underlie this fundamental political divide, often assessed by the Social Dominance Orientation scale (SDO), which predicts social and political attitudes related to dominance hierarchies^5^,^6^,^7^. Higher SDO scores denote a stronger drive to exacerbate social inequalities and a stronger belief in their legitimacy. It is thus a measure of an individual’s preference for hierarchy within any social system and the domination over lower-status groups. Yet, even across individuals sharing similar demographic profiles, a substantial variability in the attitude towards social dominance remains, hence implying that other sources of interindividual variability likely contribute to this essential personality dimension.

Here, we propose that the neural sensitivity to social dominance may accompany interindividual variability in the predisposition towards anti-egalitarianism. Previous findings have suggested that one’s social status might affect neural activities associated with the perception of others’ status^8^ and a cross-cultural fMRI study found that more dominant personality profiles are associated with greater mesolimbic BOLD response to dominant as compared to submissive body postures^9^. Moreover, individuals scoring higher on the SDO scale discriminate negative emotions better when expressed by high-status individuals^10^, contrary to those scoring lower on this scale (i.e. more egalitarian individuals). Finally, behavioral and neuroimaging measurements reported lower empathic responses to others’ suffering in people scoring higher on the SDO scale^11^,^12^.

Yet, the relationship between the neural sensitivity to social ranks deriving from competitive social interactions and the predisposition towards anti-egalitarianism remains to be investigated. To do so, we used fMRI to test twenty-eight participants performing a competitive task involving three players, as well as a cooperative task with a fourth player. During a learning phase analyzed elsewhere^13^, outcomes were covertly manipulated to induce a skill-based hierarchy composed of an inferior, an intermediate and a superior player (Fig. 1A). During a subsequent viewing phase (analyzed here), subjects were passively presented with the faces of each player, which enabled us to identify the brain network involved in the appraisal of social dominance ranks neutral faces, while excluding perceptual and task-related confounds (Fig. 1B). Our key hypothesis could then be tested by assessing the relationship between SDO scores and BOLD responses within this rank-sensitive brain network.

## Results

### Behavior

Behavioral analyses demonstrated that subjects were able to learn the hierarchy induced by the competition task. Two post tasks performed after the neuroimaging session demonstrated that subjects could correctly report the differences in the competitive skills of their opponents (for details, see ref. 13). Notably, an implicit recall procedure revealed that the *subjective* frequencies of winning (INF: 0.66 ± 0.16; INT: 0.51 ± 0.21; SUP: 0.38 ± 0.17; F(2, 54) = 27.8; p < 0.001) closely matched the *objective* frequencies of winning produced by the manipulation of outcomes during the competitive phase (INF: 0.63 ± 0.03 of victories for the subject, INT: 0.51 ± 0.03, SUP: 0.37 ± 0.06), although a substantial variance was observed at the individual level. Moreover, reaction times collected during the competitive induction phase revealed that subjects adapted their performance to the skills of the opponents, with slower response times when facing weaker opponents (F(2, 54) = 5.93, p = 0.005).

During the passive viewing phase itself, response times (RT) collected during the 30 ‘active’ trials were not modulated by the rank of opponent which preceded the apparition of the target green square (F(2, 52) = 0.55, p > 0.5), thereby excluding the possibility that subsequent fMRI results were driven by motor preparation processes (SUP: 0.36 ± 0.05; MID: 0.37 ± 0,05; INF: 0,37 ± 0.06, RT in seconds).

### Neural encoding of social ranks in the right pSTS and the right aDLPFC

In line with our hypotheses, we first contrasted the neural responses elicited by viewing SUP faces as compared to INF faces (Fig. 2). At the standard whole-brain statistical threshold, only two regions were significantly more engaged when facing superior players as compared to inferior players: the right dorsolateral prefrontal cortex in its anterior part (right aDLPFC; MNI peak: [18, 44, 34]; Fig. 2a) and the posterior part of the right superior temporal sulcus (pSTS; MNI peak: [51–55 10]; Fig. 2b). Although they did not reach the statistical threshold used for this whole-brain analysis, the left aDLPFC and left pSTS were also sensitive to social ranks (mirror aDLPFC ROI in the left hemisphere: t(27) = 2.21, p = 0.036; mirror pSTS ROI: t(27) = 2.67, p = 0.013).

Contrast estimates in the right anterior DLPFC and pSTS were uncorrelated across subjects, as if they were implementing distinct cognitive processes recruited differentially across subjects (r = 0.21, p = 0.29). Moreover, consistent with a previous study^14^, no brain region was more activated when facing inferior players as compared to superior players, even when lenient statistical thresholds were used (voxel-wise threshold: p < 0.005 or p < 0.05). A comparison of the activations elicited by the two remaining faces (control and self) with the activations elicited by the scrambled picture revealed that none of the aforementioned ROIs was responsive to this contrast (all p > 0.2; Fig. 2), thereby implying that their modulation by social ranks did not reflect low-level facial processing.

The comparison of self faces with the scrambled picture revealed face-sensitive activities in the occipital and fusiform cortices, including the fusiform face area (FFA), bilaterally (Fig. 3, left MNI peak: [−39, −49, −17].Right MNI peak: [39, −49, −17]). Note that these coordinates were in good agreement with those usually reported for the FFA (www.neurosynth.org/analyses/terms/fusiform%20face/). Although the activities induced by ranked and control faces were modest, presumably due to a habituation of FFA responses throughout the competitive task^15^, they were still significantly higher than those observed for the scrambled picture (ranked faces: t(27) = 2.47, p = 0.02; control face: t(27) = 2.85, p = 0.01; Fig. 3). Conversely, social ranks did not affect early face processing in the FFA: BOLD responses in this structure did not differ for ranked and control faces (t(27) = −0.56, p = 0.58) and they were insensitive to rank asymmetry (SUP > INF: t(27) = 1.56, p = 0.13).

### Relationship between the neural encoding of social ranks and social dominance orientation

In order to test our hypothesis that the brain sensitivity to social ranks is an important component of individual preferences for hierarchies, we extracted in each subject the mean parameter estimates from the two regions significantly modulated by social ranks (contrast of interest: SUP > INF). We then performed a correlation analysis between these parameter estimates and SDO scores. It revealed that the encoding of social ranks in the right anterior DLPFC to social ranks was strongly correlated with SDO scores across participants (r = 0.54, p = 0.003; Fig. 4A). We ran two additional control analyses to exclude that this correlation would simply derive from a differential memorization of competitive outcomes or from differential attention to the task in high *versus* low SDO participants. First, neither right aDLPFC activities nor SDO scores correlated with the recalled frequencies of winning for superior individuals minus those recalled for inferior opponents (mean difference: 0.29 ± 0.21; aDLPFC: r = −0.11, p = 0.59; SDO: r = 0.17, p = 0.38). Second, the absolute deviation between objective and recalled frequencies of winning pooled over all opponents did not correlate with right anterior DLPFC activity (r = −0.15, p = 0.45) or with SDO scores (r = 0.07, p = 0.67). In other words, neither encoding biases nor encoding quality during the competitive task mediated the correlation of interest.

In addition, we checked the correlation between rank sensitivity in the right aDLPFC and the following variables: age (r = 0.07), weight (r = 0.30), academic level (r = −0.065), smoking status (Fagerström; r = −0.15), alcohol intake (AUDIT^16^; r = −0.15), depressive symptoms (HAD scale^17^; r = −0.28), motivational orientation (BIS/BAS^18^; BIS: r = 0.13; BAS: r = 0.03), social desirability (Marlowe-Crown scale^19^; r = −0.11). None of these correlations reached significance (p = 0.05, uncorrected). Going further, we performed a multiple regression analysis including all those variables (plus the difference in subjective frequencies of winning) and SDO as competing regressors to explain variance in the DLPFC response to social ranks. Again, the SDO was the only significant factor (standardized beta = 0.621, p = 0.015). Finally, an item-wise rank correlation analysis demonstrated that two items were significantly related to the right anterior DLPFC contrast SUP > INF after Bonferroni correction (item 1 “Some groups of people are simply not the equals of others”: Spearman r = 0.543, p = 0.003; item 13, reverse-coded, “We should try to treat one another as equal as much as possible”: Spearman r = 0.564, p = 0.002).

Finally, in order to determine whether this robust brain-personality effect was driven by heightened right aDLPFC responses when facing superior opponents or by diminished responses when facing inferior opponents, we explored the correlation between SDO scores and parameter estimates of the contrasts “superior player > scrambled face”, “intermediate player > scrambled face” and “inferior player > scrambled face” (Fig. 4B). As expected, a general linear model including social rank as a within-subject factor (3 levels) and SDO scores as a covariate demonstrated a clear interaction between the neural sensitivity to social dominance and SDO scores (F(2, 52) = 6.5, p = 0.003). *Post-hoc* tests showed that the contrast “superior face > scrambled face” was positively and significantly correlated to the SDO scores (r = 0.40, p = 0.034), whereas no correlation was observed for the other two conditions (INT: r = 0.05, p = 0.8; INF: r = −0.13, p = 0.5). Thus, the interaction was mostly driven by heightened BOLD responses to dominant individuals in people scoring higher on the SDO scale.

## Discussion

Our findings confirm the hypothesis that variations in a personality trait predicting anti-egalitarism and the legitimization of social hierarchies are associated with the neural sensitivity to skill-based social hierarchies induced through iterated competitive interactions. The degree to which the right anterior dorsolateral prefrontal cortex discriminated superior individuals from inferior individuals was indeed strongly correlated with the social dominance orientation scale. More specifically, subjects who tended to prefer hierarchical social structures and to promote socially dominant behaviors exhibited stronger responses in the right anterior DLPFC when facing superior players.

A pioneering neuroimaging study highlighted the involvement of the DLPFC and the pSTS in encoding the social rank of others^14^ *during* a competitive task in which multiple processes related to reward expectations, attention and perception were also recruited. As a result, activations were embedded in a wide network encompassing early visual areas, the parietal cortex, the hippocampus, the ventral striatum, the precuneus and the amygdala. Since our study focused on the responses to neutral faces outside competitive, learning or rewarding contexts and used no perceptual cues such as symbols (eg. stars indicating the rank), uniforms, facial dominance features or body postures^12^,^14^,^20^,^21^,^22^,^23^,^24^, the DLPFC and the pSTS activations observed here may constitute the minimal, task-independent brain network involved in social rank perception. Predominantly right-sided, this core network may coordinate with additional areas depending on the task and/or the nature of the ongoing social interaction. In turn, the computational demands associated with specific tasks and contexts (e.g the need to make explicit judgments about social ranks, to compete or to assess the control exerted by dominant individuals) may engage a wider array of brain areas to adapt behavior accordingly^14^,^22^,^25^,^26^,^27^.

Making inferences about competence or dominance from faces is an ecologically important process. For example, voting based on first impressions of faces of candidates has been shown to predict real electoral outcomes^28^,^29^. A recent study proposed that social evaluation may entail distinct neurocognitive processes when assessing initial impressions or changing one’s mind about a candidate competence based on subsequent observations^30^. In this study, job candidates’ competence was first evaluated from photographs only and again after seeing snippets of their job interviews. Interestingly, the extent to which job interview information increased evaluations of competence was associated with right DLPFC activation. Here, we provide evidence that this region can also be task-independently engaged whenever information about social dominance ranks is available. Moreover, since SDO scores explained almost 30% of the variance observed in the modulation of right aDLPFC by social ranks, this brain structure might play a significant role in the prevalence of employment discrimination against women or ethnic minorities, which is directly related to the conservative and hierarchy-enhancing attitudes indexed by SDO scores^31^.

Political divides regarding inequalities in the access to resources, power and status do not only constitute a challenging societal issue. They also raise fundamental questions. Why do humans differ so much in their political preferences? How is it possible that complete reversals of causality occur in the perception and the justification of these preferences, with some people arguing that dominance hierarchies derive naturally from asymmetries of ability and other people arguing that these asymmetries are mostly generated by dominance hierarchies? Our results suggest that this polarization may stem from variations in the neural sensitivity to others’ skills, and more particularly to those of dominant others. Interestingly, the two SDO items most strongly related to this neural sensitivity reflected the two main features of the SDO questionnaire: (i) taking the existence of inferior groups as granted (which implies a pre-existing concern for social ranks) and (ii) denying the need to resorb social inequalities (which implies a reinforcement or a maintenance of existing dominance asymmetries). The sensitivity to dominance relationships and the tendency to exacerbate them may thus reinforce each other, until the point where hierarchy-related information becomes extremely salient and hierarchy-enhancing behaviors very prevalent at the individual level. Given that anger is often viewed as a marker of social dominance^32^, the fact that high SDO scores are associated with superior anger identification in dominant others^10^ concurs with our findings to suggest that anti-egalitarian preferences paradoxically implies higher sensitivity to unfavorable social asymmetries.

The correlation between rank sensitivity in the right anterior DLPFC and SDO scores may also reflect the exacerbated and automatic down-modulation of self-confidence when facing superior opponents. Indeed, perceived threats to one’s one dominance status or to the dominance status of one’s own in-group are known to exacerbate SDO scores^33^,^34^,^35^. Therefore, the inclination to affirm superiority over inferior individuals and the motivation to reach high positions in the social hierarchy might be envisionned as confidence-enhancing or stress-coping strategies. Consistently, emphasizing the significance of biological determinisms^36^ or gender differences^6^ in cognitive abilities – a hallmark of high SDO beliefs – may constitute a way to reduce the number of threatening comparison targets. Moreover, a vast literature on social comparison^37^ can explain why increased sensitivity to status threats would translate into a stronger desire to establish or maintain favorable dominance relationships: numerous findings indicate humans need to balance upward and downward comparisons with other members of their group in order to maintain their self-esteem within a functional range^38^,^39^,^40^. Supporting this hypothesis, the anterior DLPFC area has recently emerged as a domain-general correlate of subject confidence in the correctness of one’s own decisions and in the beliefs driving them. During both value-based and perceptual decision-making tasks, post-decisional confidence ratings are typically anti-correlated with aDLPFC activity^41^,^42^,^43^,^44^. Moreover, anodal transcranial direct current stimulation applied over the right DLPFC lowered confidence ratings in a short-term visual memory task, whereas accuracy remained constant^45^. Reciprocally, theta-burst stimulation used to disrupt neural activities in this brain structure increased confidence ratings in a cued perceptual decision-making task^46^, thereby showing that the causal link between metacognitive confidence and DLPFC activity is bidirectional. Finally, subjective social status and SDO are known to affect error monitoring – as indexed by feedback-related potentials in EEG^47^,^48^ – a process tightly related with metacognitive confidence ratings^49^.

It is still unclear to what extent the task of perceiving social status in others might be at the same time tracking the ability of subject to experience a hierarchical social role. In this sense, the preference for dominance would be related not only with the sensitivity to social hierarchy but also with the tendency to being involved and experiencing a social role in the hierarchy. When learning the ranks of others is based on direct competitions with them, victories and defeats are also informative regarding one’s own social dominance. In these cases, the medial prefrontal appears to track the status of the dominance relationship emerging between oneself and the others^13^,^50^, hence resulting in the so-called self-other mergence effect^51^. Yet, the right anterior DLPFC discussed here does not overlap with those mPFC activities, which reduces the relevance of this process in the specific context of our study.

To conclude, our data do not determine whether the anterior DLPFC plays a causal role in the formation of political attitudes, but suggest a link between the function of this brain region and the psychological traits that mediate political attitudes regarding dominance hierarchies. The renaissance of right-wing extremism and nationalism in most Western and Middle-Eastern societies makes this issue timely, as the desire to reinforce social inequalities based on group membership is a key aspect of the phenomenon. The neurocognitive bias reported here may eventually inform quantitative models used to describe and predict the complex dynamics of this changing political landscape^52^. Taking it into account may eventually help to better prevent and cope with the disruptive and violent behaviors sometimes observed in people scoring high on the Social Dominance Orientation scale^53^,^54^,^55^,^56^.

## Methods

### Participants

Twenty-eight right-handed men (mean age = 22.4 years, SD = 2.8, range 18–27) with no history of cardiovascular, neurological or psychiatric history, no ongoing medical treatment and corrected-to-normal vision were included in this study. Only men were tested because they typically show greater SDO scores than women^5^, because anti-female sexism correlates reproducibly with the SDO scale^6^ and because we aimed at keeping our experimental group as homogenous as possible. All participants gave informed consent and were paid 70 euros for their participation. The experimental design was approved by the Ethics Committee Sud-Est Lyon 2. All methods were performed in accordance with the relevant guidelines and regulations. At the time of the visit, subjects reported age, academic grade, weight and were screened for smoking status, alcohol intake, and depressive symptoms. At the very end of the experiments, participants reported recalled frequencies of winning against each opponent and completed the 16-items SDO scale^6^, the Behavioral Inhibition/Activation scale (BIS/BAS; Carver *et al*., 1994) and the social desirability scale^19^. These variables were then used to exclude potential confounds regarding the link between brain activity and social dominance orientation.

### Experiment

Following initial training, contextualization and instructions, participants entered the scanner. During scanner configuration, they further trained on the perceptual decision-making task and received feedback regarding the correctness of their responses following each trial, so that they could reach a stable level of performance prior to the beginning of the social competition period. The perceptual decision-making task required estimating as fast and accurately as possible the main direction indicated by a set of 46 non-moving arrows pointing either to the left or to the right. In order to ensure comparable subjective difficulty across subjects, the directional coherence of the (static) arrows was adjusted during training to converge around 80% of correct responses using a 3–1 staircase procedure. This perceptual task was framed as “reflecting overall cognitive ability by targeting the quality of basic perceptual, mathematic and decision-making skills”.

**Figure 1:**
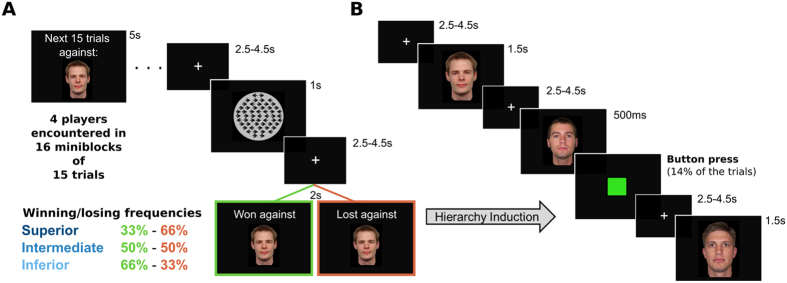
Presentation of the study. (**A**) Competitive induction phase^13^. During the 15 trials of each ‘mini-block’, subjects played against (or with) the same player in the competitive (or control) situation. The competitive task required subjects to decide as fast and accurately as possible in which direction pointed a majority of non-moving arrows displayed on the screen. Three faces were implicitly associated with 3 frequencies of winning and losing in this competitive task, thereby creating the hierarchy to be learned. (**B**) Passive presentation phase. Each opponent faces previously associated with about 33, 50 or 66% of victories for the subject was presented 30 times during 1.5s, in a random order. To control and maintain the attention of the subjects, the face was abruptly replaced by a green square in 14% of the trials, urging subjects to perform a simple button press. These trials were modelled separately.

During the competitive phase, participants were confronted to three opponents presented as real participants playing simultaneously over the internet. Competitive feedbacks were delivered in each trials (message “win against” or “lost against” over the emotionally neutral face of the current opponent see Fig. 1A). The rules of the game were explicitly defined as follows: “The fastest player wins if both responses are correct. The accurate player wins if the other gives an incorrect response. If one player doesn’t respond, then he loses automatically. If both responses are incorrect, the slowest player wins”. This latter rule was set to discourage fast-guessing responses and ensures similar number of victories and defeats across subjects. Each opponent was encountered in 4 mini-blocks of 15 trials, resulting in 60 competitive episodes in front of each. Social hierarchy was implicitly induced by covertly manipulating the frequencies of winning and losing against opponents (INF: 66% of victories, INT: 50%, SUP: 33%) and the association between faces and social ranks was counter-balanced across subjects. Note that a non-competitive social interaction with a fourth player was also included to isolate competition-specific hemodynamic signals generated at the time of the outcome. Extensive analyses about the learning phase and associated neural activities have been published elsewhere^13^.

After this competitive induction phase, subjects passively viewed the faces previously associated with different competitive skills, their own faces and a scrambled average of the four players. Each stimulus type was presented 30 times during 1.5 s in a random order. A jittered fixation cross lasting 2.5 to 4.5 s separated each display. Note that participants viewed their own face in this block because the task was framed as being done in parallel by the four other participants and had thus to remain ‘symmetrical’ until the end. In order to maintain and measure attention, participants were told to press a response button as fast as possible if a green square replaced the face displayed (the green squares appeared 500 ms after the stimulus onset). Thirty of those ‘active’ trials were randomly interleaved with the passive displays. To avoid biases, the 6 stimuli of interest were seen the same number of times (i.e., 5) in the active condition. The association between the 4 neutral faces and the social ranks were counterbalanced across subjects using a Latin square procedure, so that activities related to rank could not be explained by facial traits. The neutral faces of the 4 male players were taken from standardized database (the NimStim Set of Facial Expressions, www.macbrain.org/resources.htm). Figure 1 only displays the face of these players and not any participant’s face, for confidentiality reasons.

Once outside the scanner, social hierarchy learning was properly measured using a ‘social certainty equivalence task’ designed to assess implicitly the frequencies of winning recalled for each opponent (see ref. 13 for details). This task consisted for the subject in making a series of economic choices between a sure amount of money and a lottery offering 10€ with a probability equal to his frequency of winning against each opponent. Starting from 5€, the sure amount was adjusted downwards or upwards whenever the participant chose the lottery or not, respectively. The step size of this adjustment was divided by two after each choice (2.5, 1.25, 0.625€, etc.), so that the indifference point between the fixed lottery and the sure amount was reached rapidly (6 to 10 trials, for each opponent). The subjective probability of winning was then easily computed as the final sure amount divided by 10.

### fMRI acquisition

Imaging was conducted on a 1.5 T Siemens Sonata scanner, using an eight-channel head coil. Twenty six interleaved slices tilted relative to the anterior commissure – posterior commissure line (20–30°) were acquired per volume (field of view, 220 mm; matrix 64 × 64; voxel size, 3.4 × 3.4 × 4mm; interslice gap, 0.4 mm). Functional images were obtained using a gradient-echo echoplanar imaging (EPI) T2*-weighted sequence (TR, 2.5 s; TE, 60 ms; flip angle, 90°). Following the three fMRI runs, a high-resolution T1-weighted anatomical scan was acquired (voxel size, 1.5 × 1.5 × 1.5 mm).

### fMRI analyses

All preprocessing steps were conducted using SPM8. The first four volumes of each run were removed to allow for T1 equilibrium effects. For each subject, functional images were time-corrected, realigned, unwarped using the magnitude and phase images, and coregistered to the anatomical scan. The anatomical scan was then normalized to the MNI space using the ICBM152 template brain and the resulting transformation matrix was applied to the functional images. Finally, the normalized functional images were spatially smoothed with an 8 mm Gaussian kernel. Statistical analyses of fMRI signals were performed using a conventional two-level random-effects approach with SPM8 (http:/ www.fil.ion.ucl.ac.uk/spm).

At the first level, the general linear model (GLM) included the 6 motion parameters from the realignment step, in order to covary out potential movement-related artifacts in the BOLD signal, as well as a high-pass filter to remove low-frequency artifacts from the data (cut-off: 64 s) and a run-specific intercept. Temporal autocorrelation was modelled using an AR(1) process. The six regressors of interest (faces of SUP, INT, INF and control players, as well as subject’s face and scrambled face) were convolved with the canonical hemodynamic response function (HRF) using a boxcar of 1.5 s, matching the duration of stimulus presentation. Motor events and active trials were modelled using two additional regressors.

**Figure 2:**
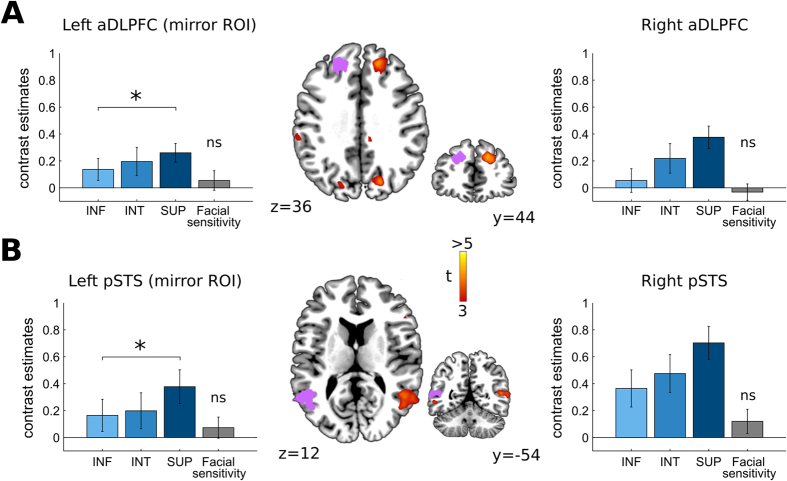
The neural representation of competitive social ranks. (**A**) Activities in the right anterior DLPFC and pSTS (bottom) modulated by social ranks at the whole-brain level (right: whole-brain analysis, p < 0.001^unc^ voxel-wise, p < 0.05^FWE^ cluster-wise; left: mirror ROI flipped along the X-axis). (**B**) A similar effect was found in the right posterior STS and in a left mirror ROI. In both regions, viewing superior player’s face (SUP, dark blue) elicited greater BOLD responses than viewing intermediate (INT, blue) and inferior players’ faces (INF, light blue). None of these regions was more activated by self and control faces as compared to scrambled faces. Contrast estimates were extracted from activation clusters (p < 0.001^unc^ voxel-wise) and their mirror counterparts. Error bars denote s.e.m.

**Figure 3:**
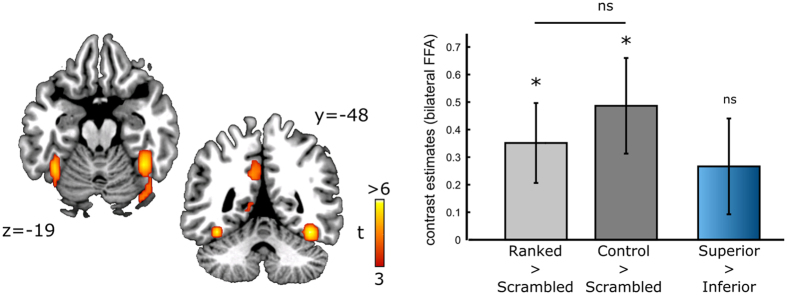
Absence of rank encoding in the fusiform face area (FFA). On the left, results of the whole-brain analysis for the contrast [self face > scrambled face] (p < 0.001^unc^, p < 0.05^FWE^) used to reveal the fusiform face area. Ranked (SUP, INT, INF) and control faces induced significantly more activity than scrambled faces in the FFA. However, the encoding of face-related activity did not differ significantly in these two conditions and no rank sensitivity (as indexed by the contrast “superior > inferior”) was observed, as shown in the right panel. Contrast estimates were extracted from 3 millimeters spheres centered on the peak voxels of FFA activity in each hemisphere and were then averaged as no laterality effect was observed. Error bars denote standard error to the mean.

**Figure 4:**
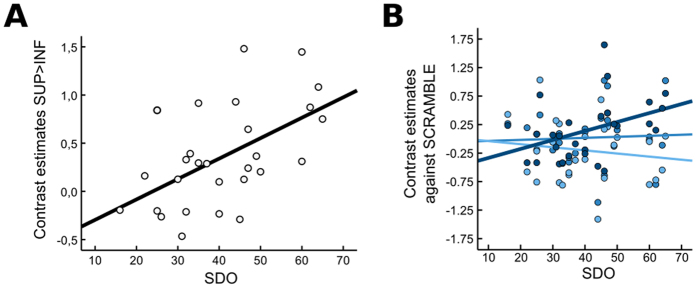
Relationship between social dominance orientation and neural sensitivity to social ranks in the right anterior DLPFC. High SDO scores reflect preference for hierarchical social structures and promotion of socially dominant behaviors. (**A**) A strong positive correlation was observed between the proneness to legitimize and reinforce social hierarchies and the activation of the right anterior DLPFC when viewing superior as compared to inferior faces, which explained 29,2% of the variance in the SDO scale (r = 0.54). (**B**) *Post-hoc*analyses indicated that this correlation was mostly driven by heightened aDLPFC responses in high SDO subjects when facing superior (dark blue) rather than intermediate (middle) or inferior (light blue) individuals. In order to maximize spatial specificity, contrast estimates were extracted from 3mm sphere center around the peak voxel of the right anterior dlPFC (Fig. 2A, right).

At the second level, inferences were based on one-sample t-tests. Statistical maps were obtained with the standard voxel-wise threshold (p < 0.001, uncorrected) and statistical inferences were drawn from activations corrected for multiple comparisons at the cluster level (p < 0.05^FWE^). All the ROI analyses reported in Figs 3 and 4 were based on 3-millimeters spheres centered around the peak voxel of interest (right anterior DLPFC: [18, 44, 34]; left FFA: [−39, −49, −17]; right FFA: [39, −49, −17]). For the mirror ROI analysis used to replicate the main effects reported Fig. 2, we simply flipped the clusters (obtained at a voxel-wise threshold of p < 0.001) of the right anterior DLPFC and posterior STS along the x-axis.

## Additional Information

**How to cite this article:** Ligneul, R. *et al*. Social brains and divides: the interplay between social dominance orientation and the neural sensitivity to hierarchical ranks. *Sci. Rep.* **7**, 45920; doi: 10.1038/srep45920 (2017).

**Publisher's note:** Springer Nature remains neutral with regard to jurisdictional claims in published maps and institutional affiliations.
